# The quality of sero-surveillance in low- and middle-income countries: status and trends through 2007

**DOI:** 10.1136/sti.2008.030593

**Published:** 2008-07-22

**Authors:** R Lyerla, E Gouws, J M Garcia-Calleja

**Affiliations:** 1 Epidemiology and Analysis Division, Joint United Nations Programme on HIV/AIDS, Geneva, Switzerland; 2 UNAIDS, Geneva, Switzerland; 3 WHO, Geneva, Switzerland

## Abstract

**Objective:**

To examine the quality of HIV sero-surveillance systems in 127 low-income and middle-income countries by 2007, as well as gaps in data needed for reliable estimates of HIV prevalence and size of populations at risk for infection.

**Methods:**

The quality of countries’ surveillance systems was scored using information from 2001 through 2007. Sero-surveillance data were compiled from the US Census Bureau’s HIV/AIDS Surveillance Database, from countries’ national HIV surveillance reports available to UNAIDS, from demographic and health survey (DHS) data, from the scientific literature and from countries’ Estimation and Projection Programme (EPP) data files. The quality of systems was scored according to the classification of the epidemic in each country (generalised, concentrated or low-level).

**Result:**

The number of countries categorised as fully functioning in 2007 was 40. 43 countries were identified as partially functioning while 44 were categorised as poorly functioning. Low scores were most often attributed to a lack of recent data or data from appropriate risk groups.

**Conclusion:**

Many countries still have poorly functioning surveillance systems. The inclusion of HIV testing in national population-based surveys in recent years has resulted in some countries with generalised epidemics receiving higher coverage scores, but many countries with concentrated or low-level epidemics continue to lack data on high-risk populations.

Tracking and monitoring the HIV epidemic remains heavily dependent upon the quality of national surveillance. As noted in earlier reports,^1^ ^2^ one purpose of HIV surveillance is to determine the magnitude of the epidemic. Twenty-five years since the start of the epidemic, a more important purpose is to track the changes or trends in the epidemic over time. Assessing trends in HIV prevalence is important for assessing the demographic impact of the epidemic, for predicting the future course of the epidemic, for determining resource allocation, as well as for monitoring the impact of interventions and country responses.

In generalised epidemics, sentinel HIV surveillance among the general population can provide essential information for planning care and support and for indicating the success of the current response. Surveillance based on women attending antenatal clinics can be used to assess trends in HIV prevalence over time. Antenatal clinic surveillance has also been used to estimate the population levels of HIV.^3^

While countries with concentrated or low-level epidemics often rely on case reporting to track their epidemics, surveillance systems that rely solely on AIDS or HIV case reporting are not effective because of the long incubation period between infection and the development of AIDS, and because people who are infected with HIV are often not detected until they become sick with AIDS. In addition, as noted by other authors,^1^ ^2^ in low-income and middle-income countries, health infrastructure is often not developed well enough to allow the levels of completeness that are required to make AIDS or HIV case reporting a reliable measure of existing levels or trends in the epidemic.

In countries with generalised epidemics, sentinel surveillance of pregnant women attending public health antenatal clinics has been recommended for monitoring the course of the epidemic since the mid-1980s.^4^ Since 2000, UNAIDS and the World Health Organization have promoted the use of second-generation surveillance tools^5^ to help countries understand their respective epidemics through the collection and analysis of surveillance data from different sources. This information can improve the ability in a country to monitor trends in HIV prevalence as well as to monitor the impact of the epidemic.

Since the quality of surveillance in Western Europe, the United States, Canada, Australia and other high-income countries have been analysed and reported elsewhere,^6^^–^9 this paper reanalyses the quality of HIV surveillance systems in low-income and middle-income countries only. Quality of surveillance in low-income and middle-income countries has been assessed before, in 1999 and 2002,^1^ ^2^ utilising data almost exclusively from the HIV surveillance database of the United States Census Bureau and country reports on HIV and AIDS available at the time to WHO and UNAIDS. The data produced by these systems are often the basis for the estimates of HIV prevalence and impact produced by WHO and UNAIDS.^10^ These estimates are then used in turn to develop outcomes related to the disease, such as mortality, orphans, impact of treatment.^11^ The paper will not review the methods used to produce estimates of HIV prevalence, which are presented elsewhere in this supplement, nor will it review other aspects (such as the impact of stigma, access to services or rates of refusal) of surveillance related to HIV.

This main objective of this paper is to score the quality of surveillance systems in low-income and middle-income countries and to identify gaps in the data that are needed to guide future research and infrastructure development.

## METHODS

### Data sources

Data on surveillance of HIV in countries come from a number of sources. In addition to the HIV surveillance database from the United States Census Bureau, (http://www.census.gov/ipc/www/hivaidsn.html), all countries participating in the UNAIDS/WHO bi-annual training workshops on HIV estimation methods were asked to provide up-to-date information on the surveillance activities and reports in their countries. Additionally, all participants were followed up by email in which they were provided with a country score from the last round of assessment in 2004, a copy of all documents used to develop the last score and that have been received from the countries since then, and a request to provide more recent documents should they exist. Finally, literature searches were conducted for recent studies on high-risk populations.

### Epidemic classification

The countries were classified according to the state of the HIV epidemic: low-level, concentrated or generalised. These definitions were initially based on epidemic levels in countries and are used to determine the most appropriate surveillance activities needed in countries—that is, identifying which data and population groups need to be included in surveillance.^5^ Briefly, in countries with low-level epidemics, HIV prevalence has not consistently exceeded 5% in any subpopulation whose behaviour places them at highest risk for infection. At this level of the epidemic, HIV surveillance should be carried out in the groups at highest risk in the country.

An epidemic is referred to as concentrated if the estimated HIV prevalence is consistently over 5% in at least one subpopulation at highest risk of infection, and has a prevalence below 1% in the general adult population (age 15–49 years) in urban areas. At this level of the epidemic, surveillance should continue in the groups at highest risk and surveillance should be started in the general populations in urban areas.

In countries with generalised epidemics, HIV prevalence is firmly established in the general adult population, and HIV surveillance activities should be conducted among adults in the general population in urban as well as rural areas

In countries with generalised epidemics, HIV prevalence data from pregnant women attending antenatal clinics have been used to monitor epidemic trends in the general adult population.^12^ While antenatal clinic surveillance only provides information on women of reproductive age, population-based or community-based studies which compare prevalence in samples of men and women in the general population have been used to calibrate the relation between this sample and the general population. In recent years, many countries have also included HIV testing in national population-based household surveys in an attempt to obtain geographically more representative data from adult populations.

### Defining the quality of sentinel surveillance systems

In the past, coding schemes representing four dimensions related to the quality of the surveillance system were developed by Walker *et al*.^1^ These dimensions, also used to score quality in this paper, are:

- Frequency and timeliness of data collection;
- Appropriateness of populations under surveillance;
- Consistency of the sites/location and groups measured over time; and
- Coverage/representativeness of the groups for the adult populations.

### Scoring surveillance quality in countries with generalised epidemics

#### Initial coding of the data

A spreadsheet was developed for each country to record available data for each year during the time period of interest (2001–7) (frequency and timeliness), the type of data available (appropriateness of populations under surveillance), consistency of the sites included in surveillance and the representativeness of the sites. Different spreadsheets were used for low level/concentrated epidemics and generalised epidemics, with the low level/concentrated epidemic spreadsheets allowing the reviewer to identify which high-risk populations had been included in surveillance.

#### Scoring sentinel surveillance quality

Scores were computed for each of the four dimensions of quality, which were then combined to create an overall score of the quality of the surveillance system for each country included in this process.

#### Frequency and timeliness

The scoring for frequency and timeliness remained a simple arithmetic calculation. The number of times national sentinel surveillance or a national population-based HIV prevalence survey had been conducted between 2001 and 2007 was counted (range 0 to 7). This total was then divided by the potential maximum (7) to obtain a ratio between 0 and 1. Since frequency was deemed important for trend analysis, this value was then doubled. Countries where data had been collected in the last two years were given an additional 1, others 0 for timeliness. The sum of these two values (with a maximum of 3) was used as the overall measure of frequency and timeliness.

#### Appropriateness

An appropriate surveillance system was defined as one in which data had been collected in the last seven years in urban as well as rural sites from antenatal clinic surveillance. In this round of assessment, if a national population-based survey had been conducted between 2001 and 2007, it was also counted as “appropriate”. Countries with an appropriate system were scored as 1, all others were scored as 0.

#### Consistency

Scoring for consistency was a judgment made by the reviewer of the country data. As stated by other authors,^1^ ^2^ trends in the epidemic cannot be accurately measured if data are not collected repeatedly from the same sites over time. The score for consistency was made on a 3-point scale, with 0 representing no pattern of consistency in urban and rural sites from antenatal clinic surveillance, a score of 1 was given when there was some repetition in sites and where it could be determined that the data provided some information about trends, and a score of 2 was given for surveillance systems where a clear pattern of consistency was evident. This value was divided by 2 (the maximum score) to obtain a ratio between 0 and 1. A score of 1 suggests that there was sufficient consistency among sites to measure trends in the epidemic among pregnant women in both urban and rural areas. Judgments of consistency were made independently by two reviewers.

#### Coverage

Coverage was scored differently in this round of assessment compared to the assessment in 2004. Coverage was scored on a 4-point scale, 0–3, with 0 reflecting poor coverage, 1 reflecting some evidence of an increasing surveillance capacity, 2 if the surveillance system in antenatal clinics is fully representational in urban and rural areas, and 3 if a DHS or national population-based survey with HIV testing was conducted in addition to sentinel surveillance. This value was then divided by 3 (the maximum score) to obtain a ratio between 0 and 1.

#### Overall quality of the surveillance systems in generalised epidemics

The overall quality of the surveillance system was determined by the sum of the scores for each of the four dimensions. The sum of these four dimensions ranged from 0 to 6, with frequency and timeliness contributing to half of the total. As in the past, three categories of quality were used; fully implemented, partially implemented and poorly implemented. Countries scoring more than 4 out of 6 were regarded to be fully functioning, representing surveillance systems that were timely, frequent, appropriate and representative. Countries scoring from 2 to 3.9 were rated as partially implemented, characterised by having some features of a high-quality system, but not all. Many of these countries needed more frequent surveillance. Countries scoring lower than 2 were characterised as poorly functioning in which none of the countries were determined to have sufficient data to track the trends in the epidemic. As a result of this analysis, gaps in surveillance systems can be identified and utilised to enhance country-specific surveillance systems.

### Scoring surveillance quality in countries with concentrated and low-level epidemics

**Figure 1:**
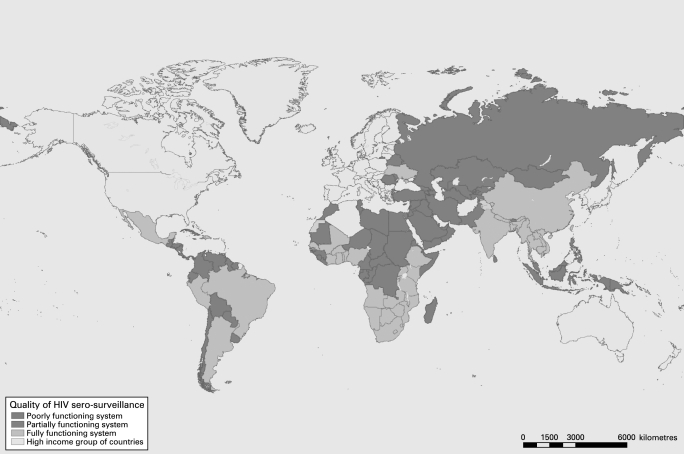
Quality of HIV surveillance systems, 2007.

Scoring quality of surveillance systems in countries with low-level or concentrated epidemics was different from scoring for countries with generalised epidemics (fig 1). In countries with low-level or concentrated epidemics, the risk of infection is concentrated in groups that report behaviours associated with HIV infection. Information needed for monitoring the trends in the epidemic in these countries must therefore come from groups at higher risk for infection.

#### Initial coding of data

A separate spreadsheet was used for countries with low-level or concentrated epidemics. Surveillance systems in these countries were assessed based on the presence of data from four of the groups most at risk for infection: commercial sex workers (CSW), clients of CSW, men who have sex with men (MSM) and injecting drug users (IDUs). Data were also collected on surveillance conducted among patients seeking care for sexually transmitted infections (STI), which might serve as a proxy for high-risk individuals in some settings. For each category, the years in which data were collected were also recorded.

#### Scoring surveillance system quality

As for countries with generalised epidemics, quality of surveillance systems in low-level or concentrated epidemics were scored based on the four dimensions outlined above. *Frequency* was scored as described above, as was *timeliness*, with a summed score ranging between 0 and 2, but assessing surveillance systems among population at higher risk for infection over time.

*Appropriateness and coverage* were combined and defined by those populations that had been under surveillance. In low-level and concentrated epidemics it has been determined that it is not necessary to be concerned about full geographical coverage, since these populations are often clustered in urban areas.^13^^–^15 Therefore, if all high-risk populations were reported on, full scores (with a maximum of 4) were awarded in appropriateness and coverage. If some high-risk populations were not considered, lower scores were awarded. For countries to receive the highest score (3) at least three risk groups and pregnant women in urban areas would have to be included in surveillance. For a score of 3, the surveillance system must include data from three high-risk groups, MSM, IDU and CSW; for a score of 2, a country surveillance system would include data from only one high-risk group and some other source (for example, antenatal clinics, prisons, migrant population, etc); for inclusion of only one data source, countries received a score of 1; all other countries received a 0 score. This score was divided by the maximum (4) to obtain a ratio between 0 and 1.

For low-level epidemics, data should be collected from all groups at risk for infection in order to be considered fully implemented. For countries with concentrated epidemics, there should also be surveillance among pregnant women in urban areas.

As initially proposed by Walker *et al*,^1^ consistency was again judged by considering the reported sites over time. First, a measure of consistency was estimated: if the same sites were consistently included over time, 1 point was given, 2 was given for some consistency and 3 was given for no or very little consistent use of sites. Second, the number of times data had been collected in any of the higher-risk populations over the last seven years (with a maximum of 7) was counted and divided by 7, similar to the frequency score above. In this round of scoring, the overall consistency score was then calculated by dividing the frequency score by the measure of consistency (1, 2 or 3). Those with greatest consistency received the largest score, those with the weakest, the smallest. The overall score could range between 0 and 1.

#### Overall quality of the sentinel surveillance systems—concentrated and low-level epidemics

As in the case of countries with generalised epidemics, a summary score was developed by combining different dimensions of surveillance, with the total ranging between 0 and 4. Different cut-off scores were used for concentrated and low-level epidemics. For both concentrated and low-level epidemics, poorly functioning surveillance systems received a score of 0 to 1.6, partially functioning systems were scored 1.7 to 3.3 and fully functioning were score 3.4 or higher.

## RESULTS

Results of the quality of HIV surveillance in 127 low-income and middle-income countries are presented in table 1 by geographical region. As shown in the table, 40 countries are categorised as having generalised epidemics, 49 countries have concentrated epidemics and 38 counties in this assessment have a low-level HIV epidemic.

**Table 1:** Surveillance system quality score by country, 2007

Country	State of epidemic	Quality rating
**Caribbean**		
Bahamas	C	2
Barbados	C	1
Cuba	C	1
Dominican Republic	C	3
Haiti	G	3
Jamaica	C	1
Trinidad and Tobago	C	1
**East Asia and Pacific**		
China	C	3
Fiji	L	1
Mongolia	L	1
Papua New Guinea	C	2
**Eastern Europe and Central Asia**		
Armenia	C	2
Azerbaijan	L	1
Belarus	C	2
Bosnia and Herzegovina	L	1
Georgia	L	2
Kazakhstan	C	2
Kyrgyzstan	L	2
Republic of Moldova	C	2
Romania	L	1
Russian Federation	C	2
Tajikistan	L	2
Turkmenistan	L	1
Ukraine	C	3
Uzbekistan	C	2
**Latin America**		
Argentina	C	3
Belize	C	2
Bolivia	L	1
Brazil	C	3
Chile	C	1
Colombia	C	2
Costa Rica	C	1
Ecuador	C	2
El Salvador	C	2
Guatemala	C	2
Guyana	C	2
Honduras	C	2
Mexico	C	3
Nicaragua	C	1
Panama	C	1
Paraguay	C	2
Peru	C	3
Suriname	C	2
Uruguay	C	2
Venezuela	C	1
**North Africa and Middle East**		
Algeria	L	1
Bahrain	L	1
Cyprus	L	1
Egypt	L	2
Iraq	L	1
Israel	C	1
Jordan	L	1
Kuwait	L	1
Lebanon	L	1
Libyan Arab Jamahiriya	L	1
Morocco	L	2
Oman	L	1
Qatar	L	1
Saudi Arabia	L	1
Sudan	G	1
Syrian Arab Republic	L	1
Tunisia	L	1
Turkey	L	1
United Arab Emirates	L	1
Yemen	L	1
**South and South-East Asia**		
Afghanistan	L	1
Bangladesh	L	3
Bhutan	L	1
Brunei Darussalam	L	1
Cambodia	C	3
India	C	3
Indonesia	C	2
Iran (Islamic Republic of)	L	2
Lao Peoples’ Democratic Republic	L	3
Malaysia	C	2
Maldives	L	1
Myanmar	C	3
Nepal	C	3
Pakistan	L	2
Philippines	L	2
Sri Lanka	L	2
Thailand	C	3
Vietnam	C	3
**Sub-Saharan Africa**		
Angola	G	3
Benin	G	3
Botswana	G	3
Burkina Faso	G	3
Burundi	G	3
Cameroon	G	2
Central African Republic	G	2
Chad	G	2
Comores	C	1
Congo	G	2
Cote d’Ivoire	G	3
Democratic Republic of Congo	G	2
Djibouti	G	2
Equatorial Guinea	G	2
Eritrea	G	2
Ethiopia	G	3
Gabon	G	2
Gambia	G	2
Ghana	G	3
Guinea	G	2
Guinea-Bissau	G	1
Kenya	G	3
Lesotho	G	3
Liberia	G	2
Madagascar	C	1
Malawi	G	3
Mali	G	3
Mauritania	C	1
Mauritius	C	1
Mozambique	G	3
Namibia	G	3
Niger	G	2
Nigeria	G	3
Rwanda	G	3
Senegal	C	3
Sierra Leone	G	2
Somalia	C	1
South Africa	G	3
Swaziland	G	3
Togo	G	3
Uganda	G	3
United Republic of Tanzania	G	3
Zambia	G	3
Zimbabwe	G	3

Among the 127 countries whose surveillance systems were rated, 40 countries were judged to have fully functioning surveillance systems; 43 have partially functioning systems and 44 were rated as having poorly functioning or non-existent sentinel surveillance systems.

### Sub-Saharan Africa

This region is the most affected by the HIV pandemic.^10^ Among the 44 countries scored in this region, all but six have generalised epidemics. Twenty-four countries have systems that would be categorised as fully functioning. Many of the countries in the region have conducted national population-based surveys in which HIV testing has been included, which are often used to adjust national HIV prevalence estimates based on ANC sentinel surveillance data. A discussion of this process of adjustment is presented elsewhere in this supplement.^16^ Scores in these countries were increased by additional points if a population-based survey with HIV testing had been conducted. The fully functioning system determinations were therefore influenced by the availability of population-based prevalence data. On the other hand, six countries do not have surveillance systems that could be characterised as even partially functioning. These countries, though few in number, do not have basic surveillance activities that will allow for tracking the epidemic. Fourteen countries have partially functioning surveillance systems. Countries with the highest HIV burden have the most fully functioning surveillance systems.

Surveillance efforts in Chad and Cameroon have declined considerably in the last three years. As a result, the surveillance system in these two countries is categorised as only partially functioning in this assessment. Namibia and Togo have evidence of ongoing antenatal clinic surveillance, yet have not conducted a national population-based survey, However, their antenatal clinic surveillance is consistent, appropriate and timely and, as such, these countries are determined to have fully functioning surveillance systems.

### South and South East Asia (excluding China and India)

Among the 18 countries assessed in this region, eight are characterised as having fully functioning surveillance systems. The epidemics in most of these countries are centred in high-risk populations, are mature and, as such, surveillance of infections and tracking of the epidemic are well established. Of the remaining countries, six were partially implemented and four were functioning poorly. The region as a whole has sufficient data on IDU and CSW populations, but has limited information on MSM populations.

### China

The Chinese surveillance process has improved greatly over the past decade. There are considerably more studies in the literature that address population sizes for groups at highest risk and in 2007 the national government released an estimate of HIV prevalence^17^ in the country that represented a combined estimate from all provincial-level estimation calculations. In this round of assessments, China is rated as a fully functioning surveillance system.

### India

India has historically relied on data from sentinel surveillance to monitor the epidemic. However, it has long been suggested that, in fact, there was no single epidemic in the country, but many different foci of infections that were regionally diverse. A recent national population-based survey in the country provided a more robust measure of the actual prevalence and, as such, the estimated national prevalence was lowered.^18^ ^19^ Sentinel surveillance systems have also improved significantly over time and India is rated as having a fully functioning surveillance system.

### Eastern Europe and Central Asia

The 14 countries in this region all continue to have low-level or concentrated epidemics. The epidemics in Russia and the Ukraine continue to grow, however, with Ukraine surpassing a prevalence of 1.6%.^10^ Of the countries in this region, nine have developed at least partially functioning surveillance systems. Four have poorly functioning systems, and only one, the Ukraine, has a fully functioning system. Weaknesses in this region are due in large part to the practice of utilising HIV and AIDS case-reporting, and to the requirement of this assessment methodology for longer-term tracking of the epidemic. There are more studies coming from this region on particular high-risk populations, but most of the gaps in the surveillance systems are a result of the few studies in MSM populations.

### Latin America and the Caribbean

There are 27 countries in this region, and unlike what has been reported in the past,^1^ in this round of assessment, only one country is designated as generalised, while 26 are designated as low-level or concentrated. Six are fully functioning, 11 are partially functioning and 10 are poorly functioning.

### North Africa and the Middle East

The 20 countries in this region all have low-level or concentrated epidemics. Although five countries have data available on HIV prevalence in high-risk populations, only two of these countries, Egypt and Morocco, have the quantity and quality of data required to partially track the epidemic. There is a need to conduct behavioural studies in the region to determine if the behaviours placing individuals at risk for infection are rarely present, or simply not visible.^20^ There are a few studies among CSW, but virtually none among MSM or IDU.

## DISCUSSION

The analysis of the quality of surveillance systems for HIV continues to reflect wide variations in monitoring the epidemic, within and across regions. Fewer countries in this round of assessment have been categorised as fully functioning (40 vs 48) and fewer have been categorised as poorly functioning (44 vs 57) compared to the previous round of assessment in 2004.^2^ Over 68% of the global number of HIV infections are in sub-Saharan Africa,^19^ and in those countries with the highest HIV prevalence, the surveillance systems are generally good. As such, estimates of HIV prevalence and its impact in those countries, particularly in countries that have also conducted national population-based HIV surveys, are generally robust and of good quality. With better surveillance data, HIV estimations in these countries have improved over time and are continually adjusted as data and assumptions are updated,^10^ providing a more robust picture of the global burden of disease. Indeed, the quality of surveillance in many countries in this region has improved owing to expanded sentinel surveillance systems as well as the availability of information from national population-based surveys in which HIV testing has been included.

Surveillance systems judged to be of higher quality are also found in South and South East Asia. As in sub-Saharan Africa, the epidemics in some countries in this region are older and more mature than in other regions and surveillance systems in the majority of these countries are well functioning. Only about a third of the surveillance systems in Latin America and the Caribbean have sufficient information to be a fully functioning surveillance system. In the rest of the countries considered in North Africa and the Middle East, and in Eastern Europe and Central Africa, surveillance systems are either partially functioning or not functioning well at all. The exception in Eastern Europe was the Ukraine with a well functioning surveillance system, particularly among IDU.

The results of this analysis reveal general overall weaknesses in most (68%) of the evaluated countries’ surveillance systems. The number of countries with low-level or concentrated epidemics with studies considering MSM is limited almost exclusively to countries in Latin America and South Asia. This is also true for CSW. This may represent the true nature of the disease burden, the focus of prevention interventions or may represent populations more easily accessible and accepted by the local culture. In countries in Central and Eastern Europe, the preponderance of information about the epidemic comes from studies focusing on IDU.

Additionally, even in those countries where data exist for the populations most at risk of infection, consistent data collection over time does not, and trends in the epidemic are difficult if not impossible to monitor. Though the methodology used in this assessment attempted to address representativeness and coverage of surveillance, the actual size of high-risk populations remains difficult to quantify. Most HIV prevalence data in these high-risk populations are found in specific studies with limited sample size and limited sampling frames. Accurate extrapolation of the results of these studies to the specific high-risk population would require an estimate of the population size. The value of data from studies such as these would be enhanced by estimations of the size of the high-risk populations. Further, just as correction factors have been suggested for prevalence estimates based on ANC data using data from population-based surveys, studies are needed to determine if such adjustments are needed for estimations among high-risk populations (that is, Workbook or EPP).^21^ ^22^

The analysis presented here has several limitations that must be considered. First, there is the possibility that some data in countries have not been considered in this analysis. Every effort has been made to ensure that available data have been identified by contacting national epidemiologists from each country and requesting the most recent data available. The scope of the data considered appropriate for the analysis in low-level or concentrated epidemics was limited to data on high-risk populations, and for the analysis in generalised epidemics was focused on antenatal clinic surveillance and population-based surveys that include HIV testing. If a country uses data from other sources for monitoring the epidemic, such as data related to blood screening, those data were not captured here. Additionally, publications were considered in English, French, Spanish and Russian. Other languages were not considered.

Surveillance of infections such as HIV is of course problematic because of the asymptomatic nature of the infection for most of the duration of the infection. For many individuals in countries with low-level and concentrated epidemics, barriers to testing (such as access and stigma) make it difficult to adequately assess the true burden of infection in certain populations.^23^ However, with the increase in the availability of treatment, an increase in the number of people undergoing counselling and testing and an increase in the number of identified cases might be expected.

This paper considered the most recent data available for the last seven years in assessing the quality of surveillance systems for monitoring the HIV epidemic. In countries with the largest disease burden, surveillance has continued to improve over time, and the addition of large population-based HIV prevalence surveys in these countries has greatly enhanced the reliability of the data. In many other countries, specifically those with low-level and concentrated epidemics, the quality of data has also improved, though many countries still lack the consistency required to follow trends over time in these high-risk populations. There are gaps in some countries’ data on high-risk populations, and behavioural data are generally scarce. Estimating the size of high-risk populations and the frequency of exposure to HIV remains a difficult task.

**Key messages**

- The quality of HIV surveillance seems to be improving.
- However, some countries still lack the necessary data to accurately assess the course of the epidemic. Trend analyses will be difficult.
- Many countries with low-level and concentrated epidemics could improve their surveillance by collecting data from all groups at risk for infection.

## References

[1] Walker N Garcia-Calleja JM Heaton L Epidemiological analysis of the quality of HIV sero-surveillance in the world: how well do we track the epidemic? AIDS 2001;15:1545–54 11504987 10.1097/00002030-200108170-00012

[2] Garcia-Calleja JM EZaniewski E Ghys PD A global analysis of trends in the quality of HIV sero-surveillance. Sex Transm Infect 2004;80 (Suppl 1):i25–30 15249696 10.1136/sti.2004.010298 PMC1765843

[3] UNAIDS/WHO Guidelines for measuring national HIV prevalence in population-based surveys, ed. UWWGoGHAS Surveillance. Geneva: World Health Organization, 2005:67

[4] Chin J Mann J Global surveillance and forecasting of AIDS. Bull World Health Organ 1989;67:1–11 2706724 PMC2491210

[5] UNAIDS/WHO Guidelines for second generation HIV surveillance. UNAIDS/WHO Working Group on global HIV/AIDS and STI surveillance, 2000

[6] Public Health Agency of Canada HIV and AIDS in Canada. Surveillance and Risk Assessment Division and Centre for Infectious Disease Prevention and Control. Toronto: Health Canada, 2007

[7] Centers for Disease Control and Prevention HIV/AIDS surveillance report, 2005. Atlanta: US Department of Health and Human Services, Centers for Disease Control and Prevention, 2007

[8] National Centre in HIV Epidemiology and Clinical Research HIV/AIDS, viral hepatitis and sexually transmitted infections in Australia. Annual surveillance report 2007. Sydney, NSW and Canberra ACT: National Centre in HIV Epidemiology and Clinical Research, Australian Institute of Health and Welfare, 2007

[9] Japanese National AIDS Surveillance Committee 2006 Annual report. Tokyo: Specific Disease Control Division, The Ministry of Health, Labour and Welfare, 2006

[10] UNAIDS EpiUpdate 2006. Joint United Nations Programme on HIV/AIDS (UNAIDS), Geneva, 2006

[11] Stover J Walker N Grassly NC Projecting the demographic impact of AIDS and the number of people in need of treatment: updates to the Spectrum projection package. Sex Transm Infect 2006;82 (Suppl 3):iii45–50 16735293 10.1136/sti.2006.020172 PMC2576732

[12] World Health Organization Sentinel surveillance for HIV infection. WHO/GPA/DIR/88.8. Geneva: WHO, 1988

[13] Vandepitte J Lyerla R Dallabetta G Estimates of the number of female sex workers in different regions of the world. Sex Transm Infect 2006;82 (Suppl 3):iii18–25 16735288 10.1136/sti.2006.020081 PMC2576726

[14] Caceres C Konda K Pechenyet M Estimating the number of men who have sex with men in low and middle income countries. Sex Transm Infect 2006;82 (Suppl 3):iii3–9 16735290 10.1136/sti.2005.019489 PMC2576725

[15] Aceijas C Global overview of injecting drug use and HIV infection among injecting drug users. AIDS 2004;18:2295–303 15577542 10.1097/00002030-200411190-00010

[16] Gouws E Mishra V Fowler T Comparison of adult HIV prevalence from national population-based surveys and antenatal clinc surveillance in countries with generalized epidemics: implications for calibrating surveillance data. Sex Transm Infect 2008;84 Suppl I:i17–i23 18647861 10.1136/sti.2008.030452 PMC2569190

[17] State Council AIDS Working Committee Office and UN Theme Group on AIDS in China A joint assessment of HIV/AIDS prevention, treatment and care in China (2007). Beijing: Ministry of Health, 2007

[18] International Institute for Population Sciences (IIPS), Macro International National Family Health Survey (NFHS-3), 2005–06: India. IIPS. Mumbai, 2007

[19] UNAIDS AIDS epidemic update 2007. Joint United Nations Programme on HIV/AIDS (UNAIDS), Geneva Switzerland, 2007

[20] Jenkins C Vulnerability to HIV/AIDS in the Middle East and North Africa: a socio-epidemiology overview. Twenty-Fifth International AIDS Conference, Satellite Meeting of Global Researchers of HIV/AIDS in the Middle East and North Africa Region. Bangkok, Thailand, 2004

[21] Lyerla R Gouws E García-Calleja JM The 2005 Workbook: an improved tool for estimating HIV prevalence in countries with low-level and concentrated epidemics. Sex Transm Infect 2006;82 (Suppl 3):iii41–44 16735292 10.1136/sti.2006.020198 PMC2576736

[22] Brown T Grassly NC Garnett G Improving projections at the country level: the UNAIDS Estimation and Projection Package 2005. Sex Transm Infect 2006;82 (Suppl 3):iii34–40 16735291 10.1136/sti.2006.020230 PMC2576727

[23] Chesney MA Smith A Critical delays in HIV testing and care: the potential role of stigma. Am Behav Sci 1999;42:1162–74

